# Ultrasound use in Kent, Surrey and Sussex Air Ambulance: a survey

**DOI:** 10.1186/1757-7241-23-S2-A18

**Published:** 2015-09-11

**Authors:** Rachel EM Tresman, Anthony Hudson, Richard Lyons, Emily McWhirter

**Affiliations:** 1 Newcastle Medical School, Newcastle University, Newcastle Upon Tyne, England; 2 Kent Surrey and Sussex Air Ambulance, Marden, England

## Introduction

Kent Surrey and Sussex (KSS) air ambulance utilize a portable ultrasound (US) scanning machine with a high frequency linear probe. Current standard operating procedure (SOP) states it should be used as an adjunct to chest assessment. Formal indications for pre-hospital US use are not yet established despite many theoretical uses in the literature.

## Aims

This study aimed firstly to assess the use of ultrasound in the unit. Secondly, to survey views on indications for pre-hospital US and finally to audit training and confidence levels within the unit.

## Methods

A survey of 17 air ambulance practitioners who attended clinical governance days was undertaken. This consisted of 9 doctors and 8 paramedics/critical care paramedics. The questionnaire was drafted with assistance from the KSS research and audit lead.

## Results

69% of clinicians estimated US use in <25% of thoracostomies and 53% estimated use of US in <50% of polytrauma cases. Reasons for not using US varied significantly; the most common being that it would not change management (39%) and a need for thoracostomy regardless (28%). Opinions varied about indications for US, the most popular being thoracic, FAST, cardiac and surgical airway. Training level varied widely; 77.8 % doctors undertook the Level 1 course and 87.5% paramedics had HEMS crew course training. Doctors had a mean confidence in performing thoracic US of 77.8%, and paramedics 67.5%.

## Discussion

Use of US is inconsistent despite inclusion in the SOP. The reason is multifactorial and it is clear from this survey that use and opinion of utility of US varies widely. However, increased formal training, support and supervision could increase use, improve confidence and aid decision making. Despite several possible applications, well designed clinical trials are needed to help define the role of pre-hospital US and courses specific to this may aid uptake of the skill.

